# Why Has Digital Contact Tracing Worked Differently in Different Countries? Comment on Cao et al. The Impact of Digital Contact Tracing Apps Overuse on Prevention of COVID-19: A Normative Activation Model Perspective. *Life* 2022, *12*, 1371

**DOI:** 10.3390/life12101592

**Published:** 2022-10-13

**Authors:** Daniele Giansanti

**Affiliations:** Centre Tisp, Istituto Superiore di Sanità, 00131 Roma, Italy; daniele.giansanti@iss.it; Tel.: +39-06-49902701

I am writing you regarding your interesting article “The Impact of Digital Contact Tracing Apps Overuse on Prevention of COVID-19: A Normative Activation Model Perspective” [1] published in the Special Issue “The Digital Health in the Pandemic Era” [2].

I found that this work particularly stimulating and gives a great added value to the field.

Specifically, I believe that this study has great merit, drawing the attention of scholars to the usefulness of digital contact tracing in the fight against COVID-19.

During the COVID-19 pandemic, many countries have used digital contact tracing apps (DCTAs) to implement contact tracing. Although the use of DCTAs has contributed to the prevention and control of COVID-19, there are doubts in academia about their actual effectiveness [1]. In your study [1], the role of DCTAs in the prevention of COVID-19 was analysed in terms of both the responsibility and inconvenience to life in a large-scale DCTA overuse environment, based on the normative activation model. I completely agree with your findings suggesting that: (a) the overuse of a DCTA activates people’s personal norms by triggering awareness of the consequences and ascription of responsibility, leading people to consistently cooperate with the government to prevent COVID-19. (b) However, the inconvenience of living with DCTA overuse weakens the effect of the awareness of consequences, ascription of responsibility and its role in influencing personal norms. (c) These effects may bear on people’s willingness to consistently cooperate with the government to prevent COVID-19. The results from your study confirmed the effectiveness of DCTAs in counteracting pandemics from a social responsibility perspective in a large-scale environment where a DCTA is used, enriching the literature on DCTA research in the COVID-19 pandemic. I am convinced that the results of your study can also help governments to design, develop and improve policies to prevent COVID-19, as well as improve DCTAs’ operating patterns.

I also, with some co-authors, faced DCTA use during the pandemic [3]. Specifically, I investigated the effectiveness of DCTA use in Italy [3]. We found that in Italy the DCTA showed both a low diffusion and a lack of capacity in the fight against COVID-19. Several factors have been identified affecting the use and diffusion of the DCTAs, for example, the strong impact of privacy issues and the digital divide [3,4,5]

Another study, based on a survey and published in the Special Issue confirmed this problem in Italy [6].

Ferretti et al. [7] had explored in the first phases of the pandemic the feasibility of protecting the population (that is, achieving transmission below the basic reproduction number) using isolation coupled with classical contact tracing by questionnaires versus algorithmic instantaneous contact tracing assisted by a mobile phone application [8]. The authors had concluded that although SARS-CoV-2 was spreading too fast to be contained by manual contact tracing, it could be controlled if this process was faster, more efficient, and increased in scale. According to the authors, the DCTAs that build a memory of proximity contacts and immediately notifies contacts of positive cases could achieve epidemic control if used by enough people. Therefore, according to these authors, DCTAs could be useful in controlling the COVID-19 epidemic. 

However, the approach based on the DCTAs [8] did not run with the same effectiveness in the world. It showed positive experiences for containing the pandemic in some countries, such as for example in China and South Korea [8] and negative experiences in other countries, such as in Italy [3,5,6].

I understand that there are a lot of factors affecting this in a positive [1] or negative way [3,4,5], and that also the national disaster culture has an important role. Based on your experience [1] and field of study I would like to open a discussion with you and have your reply.

## Data Availability

The data presented in this study are available upon request from the corresponding author. The data are not publicly available for ethical reasons.
